# Distinct enhancement of sub-bandgap photoresponse through intermediate band in high dose implanted ZnTe:O alloys

**DOI:** 10.1038/srep44399

**Published:** 2017-03-10

**Authors:** Jing Li, Jiandong Ye, Fangfang Ren, Dongming Tang, Yi Yang, Kun Tang, Shulin Gu, Rong Zhang, Youdou Zheng

**Affiliations:** 1 Jiangsu Provincial Key Laboratory of Advanced Photonic and Electronic Materials, and School of Electronic Science and Engineering, Nanjing University, Nanjing 210093, China; 2 Department of Electronic Materials Engineering, Research School of Physics and Engineering, The Australian National University, Canberra 2601, Australia

## Abstract

The demand for high efficiency intermediate band (IB) solar cells is driving efforts in producing high quality IB photovoltaic materials. Here, we demonstrate ZnTe:O highly mismatched alloys synthesized by high dose ion implantation and pulsed laser melting exhibiting optically active IB states and efficient sub-gap photoresponse, as well as investigate the effect of pulsed laser melting on the structural and optical recovery in detail. The structural evolution and vibrational dynamics indicates a significant structural recovery of ZnTe:O alloys by liquid phase epitaxy during pulsed laser melting process, but laser irradiation also aggravates the segregation of Te in ZnTe:O alloys. A distinct intermediate band located at 1.8 eV above valence band is optically activated as evidenced by photoluminescence, absorption and photoresponse characteristics. The carrier dynamics indicates that carriers in the IB electronic states have a relatively long lifetime, which is beneficial for the fast separation of carriers excited by photons with sub-gap energy and thus the improved overall conversion efficiency. The reproducible capability of implantation and laser annealing at selective area enable the realization of high efficient lateral junction solar cells, which can ensure extreme light trapping and efficient charge separation.

An intermediate band material has at least one partially filled intermediate band, by which the sub-band photons can be absorbed by exciting electrons from valence band (VB) to intermediate band (IB) and from IB to conduction band (CB), namely a two-step photon excitation^1^,^2^. In theory, solar cells using IB material can reach a photovoltaic conversion efficiency of 63% under full concentration condition^3^,^4^,^5^,^6^,^7^. Highly mismatched alloys (HMA) like GaAs:N and ZnTe:O are predicted to be a class of promising IB materials^8^,^9^. For instance, the isoelectronic O in ZnTe leads to the formation of a narrow, O-derived band of extended states as an IB within the ZnTe bandgap, which is well predicted by band-anticrossing (BAC) model and confirmed in experiments^10^. So far, ZnTe:O ternary alloys have been produced by molecular beam epitaxy (MBE), pulsed laser deposition (PLD) and metal-organic chemical vapor deposition (MOCVD) techniques, in which low solubility of oxygen seriously limited the incorporation efficiency^10^,^11^,^12^,^13^. To overcome the solubility limitation of *in-situ* doping, ion implantation is an alternative technique which has unique advantages in precise controlling of depth and dose of dopants into the host materials. The attempts to fabricate ZnTeO ternary and ZnMnTeO quaternary alloying thin films have been reported^14^,^15^. The main disadvantage of the high-dose ion implantation is the generation of massive lattice disturbances such as additional strain and high density defects, and even nanocrystallization or amorphization of the implanted layer, which consequently degrades electrical and optical properties. Annealing processes out of thermo-dynamical equilibrium are needed to recovery lattice crystallinity while activating dopants with the preserved implanted profile. Pulsed laser melting (PLM) is an effective annealing technique widely used to achieve lattice recovery in the type of processed IB materials^15^,^16^. Such process produces a melt of the implanted layer and a rapid recrystallization by liquid phase epitaxy at the radiated area within the short laser pulse, leading to a high lattice crystal quality and producing a high impurity trapping that surpasses the equilibrium solid solubility limit^17^,^18^. The degree of properties recovery and thermal diffusion of dopants are strongly dependent on the condition of pulse laser melting. In this work, the PLM processes have been optimized on O-implanted ZnTe with extra high dose to achieve high crystalline quality and formation of optically active intermediate band. The crystalline and optical recovery has been assessed by various means and the carrier dynamics related to intermediate band has been investigated in details as well.

## Results

### Structural recovery of high-dose oxygen implanted ZnTe:O.

**Figure 1:**
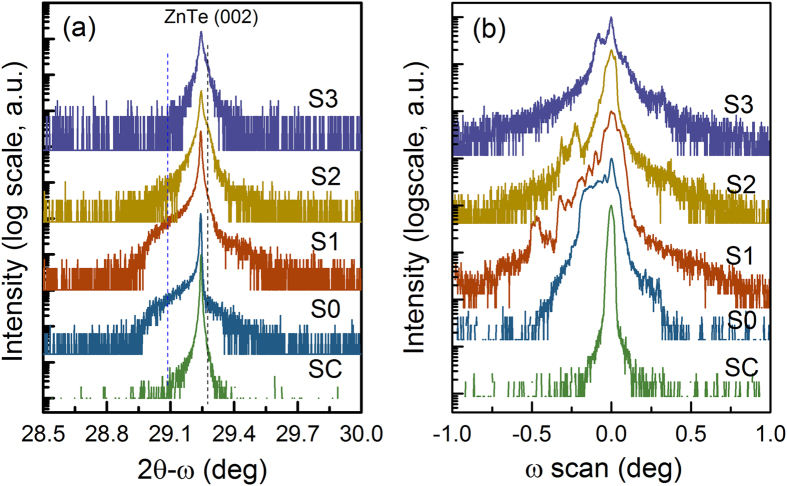
The HRXRD 2θ-ω (**a**) and ω rocking curves (**b**) of ZnTe (002) planes for ZnTe single crystal, as-implanted and PLM processed ZnTe:O samples.

Figure 1(a and b) show the HRXRD 2θ-ω and ω rocking curves of (002) plane recorded for ZnTe single crystal, as-implanted and PLM annealed ZnTeO samples, respectively. The rocking curve of ZnTe single crystal has a small full-width-at-half-maximum (FWHM) of 108 arcsec, which indicates relatively high crystalline quality. For the as-implanted sample, additional broad tails appear on both sides of single crystal ZnTe peak in 2θ-ω curves. The appearance of the low-angel peak indicates the lattice expansion in crystalline domains which have larger lattice constant than that of the single crystal, while the broad tail at high angle is related to the formation of vacancies or/and their clusters. In the ω-scan rocking curves shown in Fig. 1(b), the shoulder feature at the low-angle side is more distinguished as the ω-scan method is highly sensitive to the short-range-order scattering caused by the scattering from local Bragg diffractions of agglomerated point defects or structural grain boundaries^19^. In the rocking curve for the as-implanted sample, the thickness fringes cannot be clearly detected, which may be caused by a highly disturbed region or inhomogeneous strain distribution. High-energy implantation is expected to generate a variety of defects, such as randomly oriented mosaic blocks, dislocations, and large number of Frenkel pairs (vacancies and interstitials). As calculated by the Stopping and Range of Ions in Matter (SRIM) software, the implanted layer has extremely high vacancy concentration of about 10^22^ cm^−3^ within the projected range of 550 nm, which is beyond the critical dose for amorphization of ZnTe due to ion bombardment effect.

Upon laser annealing with energy of 0.1 J/cm^2^, the sample still exhibits similar characteristics with the as-implanted sample in 2θ-ω scan curve. It indicates that PLM processes driven by the energy of 0.1 J/cm^2^ is not sufficient to cross the threshold of melting and thus can only eliminate partial vacancy-interstitial pairs. The implanted layer still exhibits the lattice expansion with a compressive strained layer in ZnTe (001) orientation, as illustrated by a separated peak at low diffraction angle in ω-scan rocking curve in Fig. 1(b). As laser energy increases to 0.15 J/cm^2^ and above, the observed peak tails in both sides of single crystal peak in 2θ-ω scan curves disappear. It suggests that the damaged lattice is almost recovered at higher laser power. The ω-scan curves for samples S2 and S3 illustrate that the peak separation become lower, which is caused by the shrinkage of lattice expansion and also the substitution of O ions into the ZnTe lattices. Meanwhile, a noticeable peak also appears at the right side of ω-scan curves for sample S2 and S3, and shoulders are also observed in their 2θ-ω scan curves. It is resulted from the partial crystalline domains which have a smaller lattice constant than the host ZnTe materials. As named highly mismatched alloying, oxygen and tellurium have large differences in atomic size and electronegativity, and oxygen ions prefer to substitute Te sites forming the stable Zn-O with shorter bond lengths, which has been confirmed by the X-ray photoelectron spectroscopy (XPS, not shown here). In addition, it is noted that the sample annealed at 0.2 J/cm^2^ exhibits reduced intensity and broadened width of diffraction peak, indicating that excessive laser energy may lead to the bombardment effect on the implanted layer. Thus, in this work, the energy density of 0.15 J/cm^2^ is desirable for the recovery of crystalline structures.

**Figure 2:**
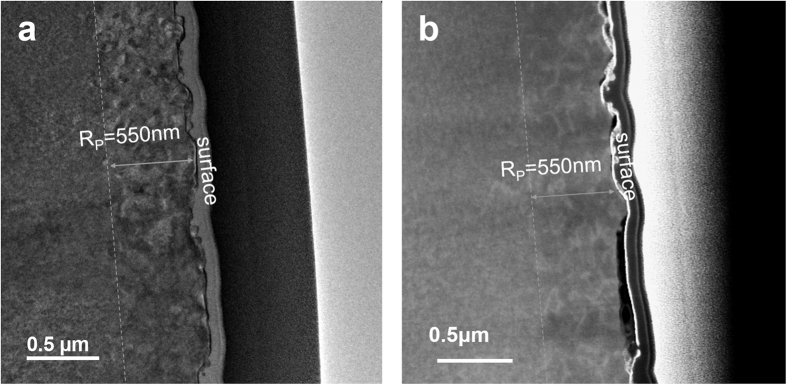
Bright-field (**a**) and HAADF (**b**) STEM cross-sectional images of sample S2.

The lattice evolution can be also evaluated directly by high resolution transmission electron microscopy (TEM). Figure 2(a and b) show a bright-field and HAADF STEM cross-sectional images, respectively, of sample S2 after PLM processing with energy density of 1.5 J/cm^2^. Overlapping black and white contrasts are visible at depths between surface and 550 nm in the bright field TEM image. This depth corresponds well to the projected range with high oxygen concentration, indicating that the implanted layer even after PLM process still consists of low density dislocations and/or dislocation loops of both vacancy and interstitial types. For HAADF STEM image, the intensity is approximately proportional to the square of the atomic number (Z-contrast imaging) and atomic column occupancy, allowing heavy atoms and vacancy clusters to be detected from the contrast features^20^. The white contrast presented in Fig. 2(b) may be resulted from the segregation of Te at the grain boundaries induced by laser irradiation during PLM processes, in agreement with the fact that Te has a larger atomic number than that of other atomic species (Z_Te_ = 52, Z_Zn_ = 30, Z_O_ = 8 and Z_ZnO_ = 38). Similarly, Shimada *et al*. has observed the Te segregation in the pulsed laser irradiated ZnTe single crystal by means of *in-situ* coherent-photon spectroscopy^21^.

**Figure 3:**
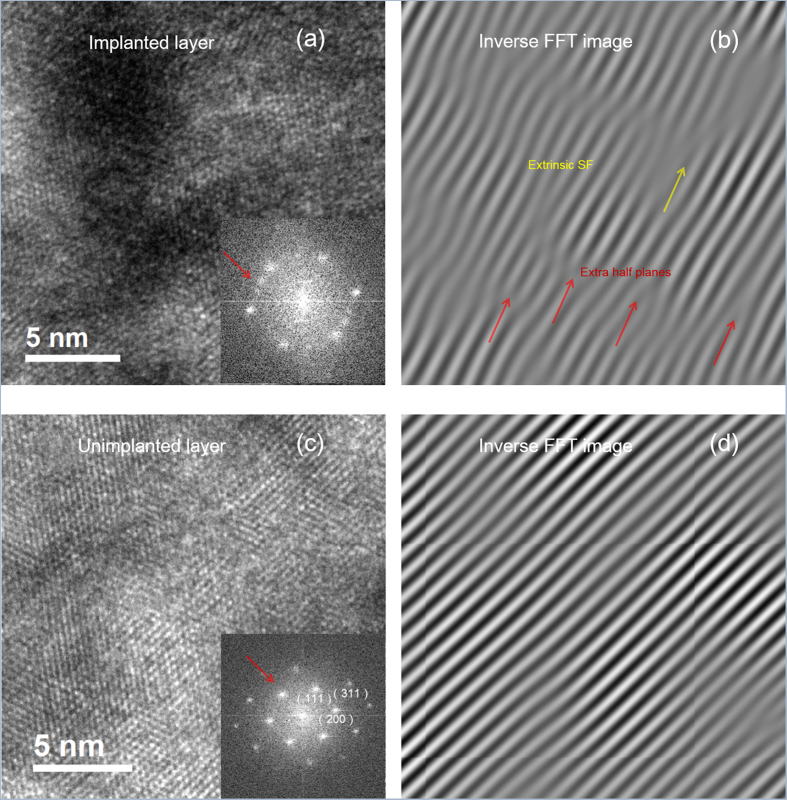
High resolution TEM image and the corresponding inset selective-area electron diffraction patterns of the implanted layer for sample S2 (**a**) and substrate (**c**); Inverse fast Fourier transformation images of the highlighted spot pairs in the patterns of implanted layer (**b**) and unimplanted layer (**d**).

High resolution TEM images with the corresponding inset selective-area electron diffraction (SAED) patterns of the implanted layer and substrates are shown in Fig. 3(a) and c), respectively. The observed excellent lattice order and the bright spots in SAED patterns illustrate the substrate has the excellent crystallinity with a typical zinc-blende phase. In contrast, the ED diagram of Fig. 3(a) exhibits extra spots distributed between the main spots corresponding to the [0–11] zone axis. This unusual pattern is an indication of the presence of dislocations and staking faults within (111) planes. In both Fig. 3(b and d), an inverse fast Fourier transformation (IFFT) analysis on the corresponding inset SAED patterns was performed. By filtering the corresponding spots with IFFT, the strain and atomic displacement maps in (111) plane are obtained and shown in Fig. 3(b and d). As compared to the substrate, lattice distortion in the form of bent planes is visible and extra half planes on the (111) planes are observed as donated by arrows, which are the sessile Frank partial edge dislocations formed by condensation of self-interstitials, such as displacement of Te on {111} clos-packed layer because of their low formation energies. The extrinsic stacking faults observed in Fig. 3(b) might be associated with local aggregation of implanted oxygen or segregation of Te. Based on the analysis of TEM results, it can be concluded that the overall crystallinity of the implanted layer has been almost recovered and a small deformation remains after PLM process, which can be ascribed to the remaining edge dislocations, dislocation loops or extrinsic stacking faults.

### Vibrational dynamics in ZnTe:O alloys

**Figure 4:**
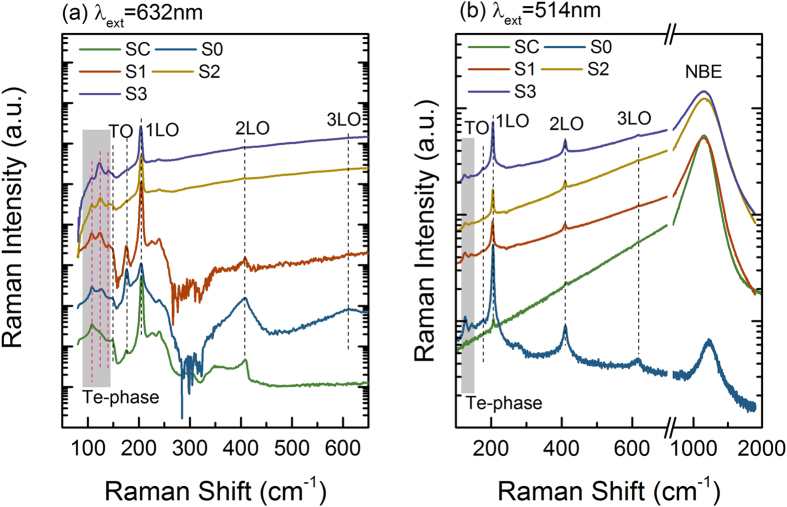
Off-resonant (**a**) and resonant Raman spectra (**b**) of ZnTe bulk single crystal, oxygen-implanted ZnTe and PLM processed ZnTe:O alloys.

To access the atomic displacement and impurity location in the ZnTe host lattice, Raman scattering characterization was conducted with a low laser density (0.2 mW) to avoid the heating effect and possible laser irradiation damage. As the bandgap of ZnTe material is about 2.26 eV, which is sandwiched between excitation energies of lasers with wavelengths of 633 nm and 514 nm, Raman scattering is under off-resonance and resonance conditions, respectively. Figure 4(a) displays off-resonance Raman spectra for series samples of ZnTe bulk single crystal, oxygen-implanted ZnTe:O and PLM processed ZnTe:O materials (S1–S3). According to the Raman selection rule, in a backscattering geometry along (100) orientation, Raman spectra mainly consist of the allowed LO branch at 204 cm^−1^ and a second-order LO (2LO) at 410 cm^−1^ for the ZnTe single crystal sample. Although the transverse optical (TO) branch induced by the deformation potential mechanism is forbidden, a week TO branch at 177 cm^−1^ and TO + 1LO zone-center phonon combination at 304 cm^−1^ are poorly resolved^22^. This could be ascribed to the presence of faceting on ZnTe surface. For the oxygen-implanted sample S0, dipole-forbidden TO mode becomes distinct accompanied by the appearance of broad high-order LO phonons, strongly implying the enhanced short-range interaction between lattice displacement and the electrons in the lattice distorted layer. It is expected that the structural disorders induced by high dose implantation result basically in the breakdown of translational symmetry of the periodic lattice, which yields a partial relaxation of the q = 0 selection rule for first- and second-order Raman scattering, and phonons from the whole Brillouin zone can be observed^22^,^23^. Moreover, implantation induced localized subgap electronic states bound to defects will act as an intermediate state for extrinsic Frohlich interaction, which leads to an additional enhancement of forbidden scattering efficiency^24^. Additional anomalous vibrational modes is also observed at the low frequencies of 110, 124, 141 cm^−1^, which have been observed in ZnTe nanowires, laser-irradiated ZnTe epilayer and CdTe^25^,^26^,^27^. These modes can be assigned to E_LO_, A_1_ and E_LO_/E_TO_ modes of the crystalline Te phase, respectively, indicating that ion implantation leads to serious Te segregation^28^. Upon PLM process with the increased laser power density, deformation potential induced TO modes gradually vanish, which is an indication of weakened short-range interaction between lattice displacement and electrons due to lattice recovery. Another evidence for reduction of lattice distortion is the enhanced and narrow LO phonon observed in PLM processed samples, in agreement with the XRD and HRTEM analysis. In comparison, the observed Te-phase related vibration modes are more distinct in the PLM processed samples, suggesting that the Te segregation is aggregated by laser irradiation, which strongly supports the localized white contrast observed in HAADF-STEM image.

The structural modification in the implanted layer has been further addressed by the resonant Raman scattering spectra as illustrated in Fig. 4(b). The Te-phase related phonons at 125 and 145 cm^−1^ are well resolved in the implanted and PLM processed samples (S1–S3) while the ZnTe single crystal sample shows no such phonons. This verifies that the aggregation of Te in the implanted layer is a result of the combination effects by high dose ion-implantation and high power laser irradiation^21^. Except for the as-implanted sample, the other samples exhibit narrow *n*LO phonons on top of strong hot luminescence bands centered at 1160 cm^−1^, which strongly implies that the optical properties of ZnTe:O are recovered with pronounced near band emissions at around 2.26 eV. In general, the electronic and optical properties are vitally sensitive to the electron-phonon interaction, which is generally originated from deformation potential and Frohlich interaction. Normally deformation potential that involves the short-range interaction between lattice displacement and electrons determines the TO Raman scattering cross section ratio, while Frohlich interaction has a long-range nature and contributes to LO Raman scattering together with deformation potential^24^. As illustrated in Fig. 4(b), the LO phonons and their overtones are greatly enhanced under resonance condition while TO modes are almost negligible, indicating that the electron (exciton)-phonon coupling is of the nature of Frohlich interaction. With the Frank-Condon approximation, the exciton-LO phonon coupling strength can be assessed by comparing the relative intensities of overtones to their fundamental Raman branch by following relationship^23^


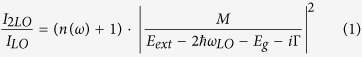


where n (ω) is the Bose factor, M is electron-phonon matrix element, E_ext_ = 2.412 eV is the incident photon energy, 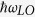 is 1LO phonon energy, E_g_ denotes the optical bandgap and Γ represents the width of the electronic states. After subtracting the PL background, the integrated intensity ratio (η) of 2LO to 1LO for the ZnTe single crystal, as-implanted and PLM processed samples (SC, S0–S3) are 1.25, 0.19, 0.27, 0.56 and 0.31, respectively. As shown in Fig. 4(b), the slight variation of optical bandgap and LO phonon energies has negligible effect on the changes of η, which are mainly determined by the phonon coherent lengths and exciton line widths (Γ), and often used to evaluate the domain sizes in ZnO, ZnTe, CdSe nanostructures^25^,^26^,^27^. For the excitons in ZnTe bulk single crystal (sample S0), electrons are more delocalized than the hole within the large crystalline domains, and the charge coupling to the lattice vibration is via long-range Frohlich interaction. The small value of η observed in the implanted sample is due to finite phonon correlation length. It is noted that high dose implantation leads to the nanocrystallization or even amorphous effect within the implanted region^17^,^18^. As a result, the superposition of wave function of electron-hole pairs is enhanced due to the localization of excitons in the nanograins, and the electric field caused by phonons can hardly polarize the excitons^23^. Namely the intensity of exciton-LO phonon coupling is weakened, resulting in the decreasing of the intensity ratio. Additionally, lattice disorder introduces abundant and dispersed localized subgap electronic states bound to defects, which act as intermediate states for extrinsic Frohlich interaction to enhance the fundamental branch of LO phonons (1LO). The PLM process undergoing liquid-phase-epitaxy mechanism is proven to recrystallize the damaged layer and reduce the lattice disorders, and consequently, the Frohlich coupling strength is enhanced, causing the increasing of intensity ratio of 2LO/1LO. However, as mentioned above, excessive laser power energy is expected to lead secondary damage to the irradiated surface region, which may weaken the electron-phonon interaction. Among PLM processed samples, sample S2 exhibits strongest near band emission and largest η, indicating that the laser power of 0.15 J/cm^2^ is proper to recover both structural and optical properties of ZnTe:O alloys.

### Optical activation and carrier dynamics of intermediate band states

**Figure 5:**
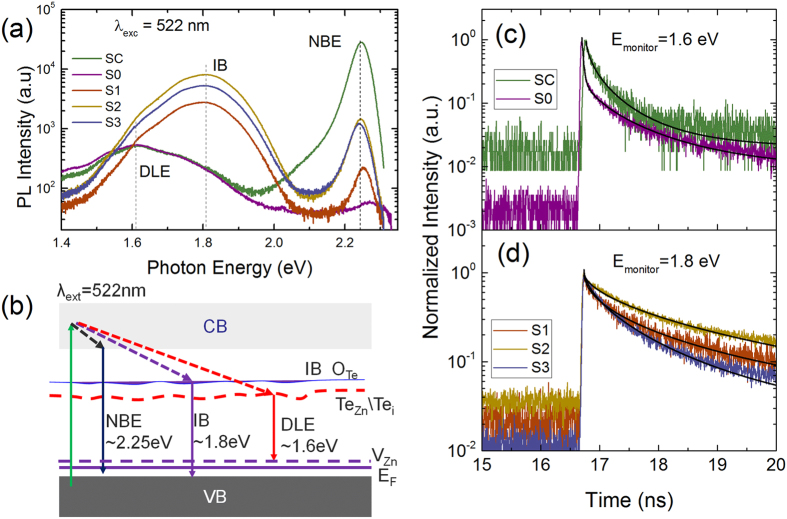
The PL spectra of ZnTe bulk single crystal, oxygen-implanted ZnTe and PLM processed ZnTe:O material (**a**); the schematic diagram for the PL process in ZnTe:O (**b**); time-resolved PL of the DLE (**c**) (for samples SC and S0) and IB (**d**) (for samples S1–S3).

Figure 5(a) displays the integrated RT-PL spectra with intensity shown in log-scale. Overall, three emission bands, including CB-VB transitions (2.24 eV), donor-acceptor transitions (1.6 eV), and transition between IB to VB (1.8 eV), are shown in Fig. 5(b). The changes in PL and time-resolved PL are related to the variation of carrier (hole) concentration, trap density and oxygen electronic states. The ZnTe single crystal exhibits a relatively strong near band edge (NBE) emission at 2.24 eV and a broad week deep level emission (DLE) at 1.61 eV, the latter is widely observed in p-type ZnTe, corresponding to the radiative recombination from donor-like Te_i_ or Te_Zn_ states to the acceptor like zinc vacancies^14^. A broad emission at 1.6 eV is dominated with a quenched weak NBE emission in the as-implanted sample owing to abundant vacancy-interstitial pairs and Te segregation induced by high dose implantation. For the PLM processed samples, the NBE emissions are recovered as a result of lattice recovery. A slight red-shift of optical bandgap of ZnTe:O approaching to that of ZnTe bulk single crystal is also observed, which is related to the released compressive strain within implanted layer, as well supported by the XRD results. The most noticeable feature is the pronounced emission around at 1.8 eV, which is attributed to excitonic emission bound to isoelectronic oxygen substituting Te atoms (O_Te_). It forms the namely IB located at 0.45 eV below the conduction band of ZnTe, as widely observed in ZnTe:O alloys and in good agreement with the energy level calculated by band anticrossing (BAC) model^8^,^9^,^10^,^11^,^12^,^13^,^14^.

The carrier dynamics involved in IB states and deep level impurity states are investigated by normalized time-resolved PL spectra as shown in Fig. 5(c and d), respectively. Notably that the PL transients, especially for the implanted sample, can be decomposed into a fast (initial) and a slow (tail) stretched exponential decay components^29^,^30^. Overall, the decay curves can be fitted well by the relationship


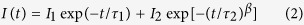


where I_1_ and I_2_ are weights of fast and slow decay components, and *β* is stretching exponent between 0 and 1. The small *β* implies a broad rate distribution due to presence of localized recombination centers or traps. The fitting results and Hall data are summarized in Table 1. Except for the implanted sample, the PL transients only exhibits a stretching exponential decay behavior (only fitted by the second term of Equation 2), which is typical for PL dynamics governed by non-radiative recombination with saturation effect of traps. As discussed above, the DLE emission at 1.6 eV is mainly originated from zinc vacancies in which hole trapping prevails. Since ZnTe single crystal has a low deep-level trap density, the fast PL decay component is hardly pronounced as the traps saturate very quickly even under low excitation power. High dose oxygen implantation (10^16^ cm^−2^) is expected to generate high density traps over 10^21^ cm^−3^ based on the SRIM simulation and the resultant higher hole concentration. In this case, trap saturation effect does not occur for sample S0 under the same low excitation power. The fast initial decay with shorter lifetime (τ_1_ < 45 ps) dominates the PL decay with a reduced weight of slow decay component. It can be understood by assuming that non-radiative recombination proceeds via both native defects and implantation induced centers with similar carrier capture cross-sections. The reduced β of 0.49 in the implanted sample also measures the effect of energetic and structure disorders such as spatial fluctuations induced by implantation.

The PL transients monitored at 1.8 eV for the IB exhibits the absence of fast initial decay and the relatively increased stretching exponent in the PLM processed samples, which implying low hole trap density owing to structural recovery and an additional recombination path for photogenerated carriers via oxygen states. Wang *et al*. have conducted the systematic investigation on generation and recombination rates at ZnTe:O IB states^31^. Consistent with their rate equation analysis, the carrier lifetimes of radiative transitions, τ_CV_ (CB-VB) and τ_IV_ (IB-VB) are inversely proportional to the product of hole concentration and their individual radiative recombination coefficient, while the relaxation time τ_CI_ (CB-IB) is determined by thermal velocity and capture cross section for electrons and the density of oxygen states, i.e. 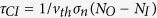
31. In the PLM processed samples, isoelectronic oxygen states with density N_O(Te)_ > 10^21^ cm^−3^ are activated by replacing Te lattice sites and implantation induced intrinsic defects are reduced greatly. Given the large electron capture cross section of oxygen states, the relaxation time (τ_CI_) from CB to IB is much lower than the lifetime τ_CV_ and τ_IV_. The fast capture into the high density oxygen states, in conjunction with the reduced CB-VB recombination rates due to low hole concentration, leads to the observed dominant IB-related emission and the relatively low intensity of CB-VB transition. The physics mechanism behind the fast relaxation is the strong interaction of extended band states in conduction band and isoelectronic oxygen states in IB as described by band-anticrossing model. Another important feature in Fig. 5(d) is that the IB-VB transitions have a longer lifetime compared to that of DLE (Table 1). This could be well explained by the localization nature of oxygen states and the reduced hole concentration in the PLM processed ZnTe:O samples. The sample S2 exhibits the strongest IB emission with the longest carrier lifetime about 1 ns. The prolonged lifetimes of carrier staying in IB states may increase the probability of transitions from IB to CB states, which is beneficial for multiphoton process and improve the ability to extract carriers in an IBSC device.

### IB mediated sub-gap absorption and photoresponse

**Figure 6:**
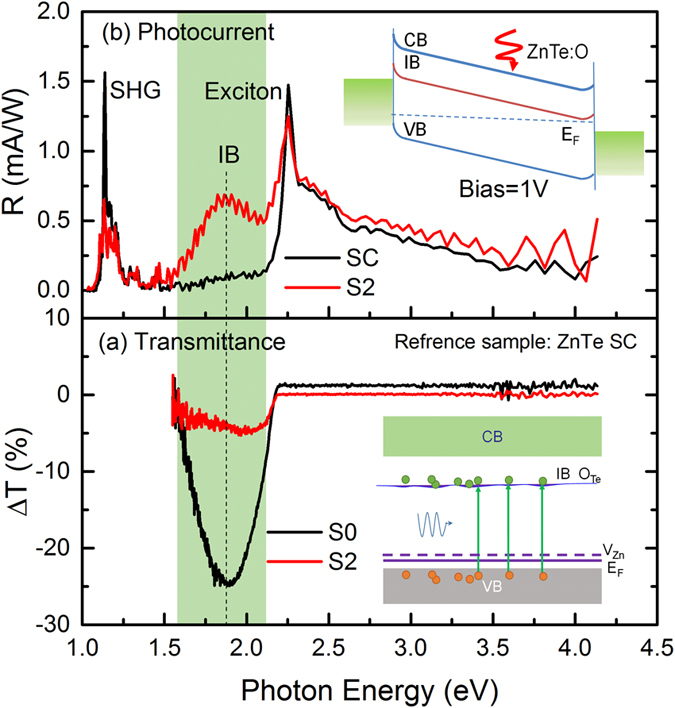
The differential transmittance spectra of ZnTe:O implanted and PLM processed samples (S0 and S2) with respect to the referenced ZnTe single crystal sample (**a**); spectral responsivity for metal-semiconductor-metal (MSM) structures with ZnTe (SC) and ZnTe:O (S2) absorber layers under the bias of 1 V (**b**).

Figure 6(a) exhibits the differential transmittance spectra of ZnTe:O implanted and PLM processed samples (S0 and S2) with respect to the referenced ZnTe single crystal. An obvious droop at 1.8 eV corresponding to IB-VB transition was observed for the as-implanted sample and dramatically deep for the PLM processed sample S2 with a large transmission difference over 20%. It is evidenced that the electrons transition from the valence band to the optically activated IB states with large electron capture cross section leads to the absorption enhancement, which is also observed in the MBE grown ZnTe:O materials and the absorption coefficient for IB was estimated exceeding 2 × 10^4^ cm^−1^ 11,32. The absorption of photons with energy of 0.45 eV is also observed in the Fig. S1 of Supplementary Materials, indicating that an electron can be excited from IB to CB before the relaxation from IB to VB taking place.

The nature of IB can be also examined by photocurrent induced by two-photo excitation process. Figure 6(b) displays the spectral responsivity for metal-semiconductor-metal (MSM) structures with ZnTe (SC) and ZnTe:O (S2) absorber layers under the bias of 1 V. For ZnTe single crystal, a sharp discrete bandedge response near 2.25 eV and a continuous broad band above bandgap with a gradual decreasing response are observed, which are contributed by exciton absorption and optical transition from VB to CB, respectively. An obvious peak around 1.13 eV (exactly half of 2.25 eV) is attributed to the exciton resonance by two-photon absorption owing to the large nonlinear second-harmonic-generation coefficient^3^. The negligible response below the bandgap also confirmed low sub-gap states in the ZnTe single crystal. In comparison, the PLM processed ZnTe:O diode exhibits a distinguished band of spectral response centered at 1.8 eV, which exactly has the same spectral overlapping with the enhanced absorption in Fig. 6(a) and IB emission in Fig. 5(a). As expected, the high electron capture cross-section and the long carrier lifetime in IB states allows the photo-excited electrons undergoing multiple transitions from the VB to IB states, and subsequently, these electrons through sub-gap transitions are driven under a bias and collected by the electrodes, giving contribution to the observed sub-gap spectral response. Note that in such M-S-M configuration, owing to the absence of electron blocking layer, the majority of photo-excited carriers in IB states are extracted directly by the electrodes, contributing to the photocurrent. From Hall measurement, the Fermi level of the PLM-processed ZnTe:O is still below the energy level of IB states, causing the small occupancy factor and thus low probability of transition from IB to CB states. Although two-photon absorption process can be verified by the near-band emission at 2.25 eV via two-photon excitation under irradiation of 633 nm and 840 nm lasers as shown in Fig. S2 of Supplementary Materials, it is hard to absorbing two photons through intermediate band under normal solar illustration. Therefore, ensuring the Fermi level across the IB is essential for realizing high optical absorption rate and improving efficiency of final IBSC devices.

## Discussion

In this work, pulsed laser melting technique has been employed to successfully recover the structural and optical properties of high dose oxygen-implanted ZnTeO highly mismatched alloys and also facilitate the formation of optically active intermediate band. Undergoing a liquid phase epitaxy mechanism, the PLM significantly reduced interstitial-vacancies defective pairs and suppressed the nanocrystallization induced by high dose implantation. The formation of an intermediate band located at 1.8 eV above valence band was confirmed by photoluminescence, absorption characteristic and photocurrent response, which is consistent with the prediction of band anti-crossing model. The carrier dynamics suggests that a relatively long lifetime of carriers in the intermediate-band electronic states is resulted from the localization nature of oxygen state and the reduction of hole concentration. By suppressing the fast recombination of electrons at IB and holes at VB, it facilitates the charge-separation process and thus improve the overall photocurrent and photovoltaic conversion efficiency. The most important is that, the reproducible capability of ion implantation and pulsed laser melting at selective area enable the realization of lateral junction of solar cells, in which directions of light absorption and charge separation are perpendicular each other and thus improve carrier collection and overall efficiency.

## Methods

### Sample preparation

**Figure 7:**
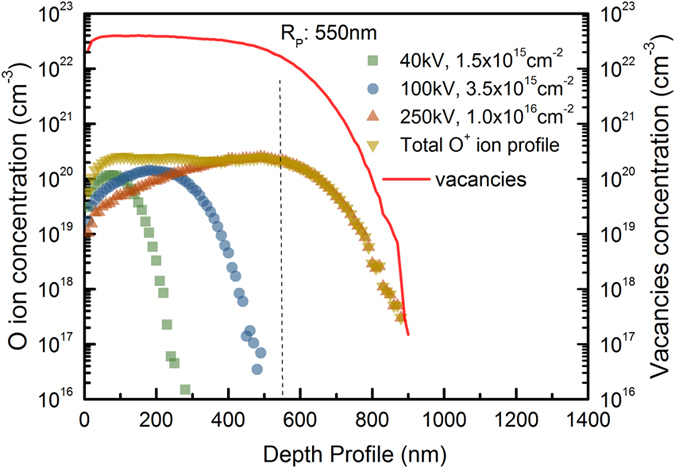
The depth profiles of O ion and vacancies caused by three-step implantation calculated by SRIM software.

The samples employed in this experiments were (001) oriented single crystal p-type ZnTe grown by chemical vapor transport method. During implantation processes, the samples were tilted 7° relative to the incident beam direction to minimize channeling effect. To obtain a uniform oxygen profile, three successive implantations at different energies and doses were carried out: accelerating voltages of 40, 100 and 250 kV with respective doses of 1.5 × 10^15^, 3.5 × 10^15^, 1.0 × 10^16^ cm^−2^ were used. Based on the calculation of SRIM software as shown in Fig. 7, an uniform distribution profiling of oxygen dopants was achieved with a projected range of 550 nm and a relatively constant oxygen concentration of 2.5 × 10^20^ cm^−3^. The corresponding oxygen mole fraction in the implanted ZnTe:O layer is 1.3%, which is beyond the solid solubility of oxygen in ZnTe due to a large difference of Te and O ions in atomic sizes. The ZnTe single crystal and oxygen as-implanted ZnTe:O samples are marked as SC and S0, respectively. After implantation, the samples were annealed by using a KrF excimer pulse laser (λ = 248 nm, pulse width = 38 ns) with different energy densities of 0.1 J/cm^2^, 0.15 J/cm^2^ and 0.2 J/cm^2^, and denoted as samples S1, S2 and S3, respectively. The KrF laser beam was homogenized yielding an irradiative area of 2 × 2 mm^2^ per pulse. The PLM processes were carried out with the laser pulsed repeating frequency of 1 Hz and pulse shots of 50 at room temperature in high vacuum condition to prevent oxidation of implanted layer by air ambient.

### Characterization

The microstructural evolution was characterized by high resolution X-ray diffraction (XRD) with a Panalytical XPert PRO MRD diffractometer. Rocking curves were measured at the ZnTe (002) reflection, which is sensitive to the strain in the vertical layer structure. A JEOL JEM-2100F scanning transmission electron microscopy (STEM)/TEM analytical electron microscope operating at 200 keV was used for bright field, high-angle annular dark field (HAADF), high resolution electron microscopy and electron diffraction measurement on the selective area. The sample used for TEM measurement was prepared by focused ion beam (FIB) equipped with a FEI Helios 600 NanoLab dual-beam system. Raman scattering spectra were recorded at room temperature using a Micro-Raman spectrometer with a Horiba JY T64000 spectrometer system in a backscattering configuration with laser lines of 632.8 nm (He-Ne laser) and 514 nm (Ar^+^ laser) as the excitation sources. The photoluminescence (PL) and time-resolved photoluminescence (TRPL) spectra were recorded at room temperature by using a micro-PL spectroscopic setup which is integrated with a charged coupled device (CCD, Princeton Instruments, PIXIS) and a time-correlated single photon counting system (TCSPC, PicoHarp 300 system). A linearly polarized laser (frequency doubled to 522 nm with 300 fs pulse width and 20.8 MHz repetition rate) was used for excitation source. The duration between laser pulses is 48 ns and the system response of PL signal decay is 45 ps. The differential transmittance spectra were recorded by a Cary 300 UV-VIS-NIR spectrophotometer with a wavelength range of 200–3200 nm and the identical ZnTe bulk single crystal was used as reference. The photoresponsivity spectra were measured by a photocurrent spectroscopic setup, which is composed of a xenon arc lamp (500 W), a Horiba i320 monochrometer, a SR830 lock-in amplifier with a chopper and a DC power supply. The photoresponsivity was calibrated by a standard Si photodiode.

## Additional Information

**How to cite this article:** Li, J. *et al*. Distinct enhancement of sub-bandgap photoresponse through intermediate band in high dose implanted ZnTe:O alloys. *Sci. Rep.* **7**, 44399; doi: 10.1038/srep44399 (2017).

**Publisher's note:** Springer Nature remains neutral with regard to jurisdictional claims in published maps and institutional affiliations.

## Supplementary Material

Supplementary Information

## Figures and Tables

**Table 1:** The list of SRIM calculated data and Hall results, and fitted parameters from TR-PL curves, of samples SC and S0–S3.

Sample	SC	S0	S1	S2	S3
Type	p	p	High-insulating	High insulating	High insulating
Conc. (cm^−3^)	2.4 × 10^15^	3.8 × 10^15^	<10^14^	<10^14^	<10^14^
O_Te_ density (cm^−3^)	—	—	~10^20^	~10^20^	~10^20^
V-I defect pair (cm^−3^)	—	~10^22^ based on SRIM simulation	—	—	—
I_1_, τ_1_	—	0.76, 24 ps	—	—	—
I_2_, τ_2_	1.01, 113 ps	0.30, 256 ps	0.99, 469 ps	0.97, 988 ps	1.05, 380 ps
β	0.65	0.49	0.57	0.58	0.56
