# Supplementary material for: Distinct enhancement of sub-bandgap photoresponse through intermediate band in high dose implanted ZnTe:O alloys
Source: Sci Rep. 2017 Mar 10;7:44399. doi: 10.1038/srep44399 (PMC5345085; doi:10.1038/srep44399)
Supplement: Supplementary Information [file srep44399-s1.docx]

Supplementary material

**Distinct enhancement of sub-bandgap photoresponse through intermediate band in high dose implanted ZnTe:O alloys**

Jing Li ^1^, Jiandong Ye[[1]](#footnote-1),2,*, Fangfang Ren^1,2*^, Dongming Tang^1^, Yi Yang^1^, Kun Tang^1^, Shulin Gu^1^, Rong Zhang^1^ and Youdou Zheng^1^

^1^ Jiangsu Provincial Key Laboratory of Advanced Photonic and Electronic Materials, and School of Electronic Science and Engineering, Nanjing University, Nanjing 210093, China

^2^ Department of Electronic Materials Engineering, Research School of Physics and Engineering, The Australian National University, Canberra 2601, Australia

**1. FTIR absorption spectra ranging from 0.4 eV to 0.8 eV**

Fig.S1. FTIR absorption spectra of samples SC, S0 and S2

The absorption properties were characterized by using a Bruker VERTEX 80v vacuum Fourier transform infrared spectroscopy (FT-IR) spectrometer. As displayed in Fig. S1, the absorption curves of SC and S0 samples have no peak around 0.45 eV, while the PLM processed sample has an obvious absorption peak located at 0.45 eV which equals with the energy gap between the intermediate band (1.8 eV above VB) and CB (2.25 eV above CB). It indicates that electrons in the oxygen-derived intermediate band states are excited into the conduction band. In conjunction with the enhanced VB-IB absorption shown in Fig. 7, it can be concluded that the introduction of intermediate band is beneficial for the enhanced absorption of photons with sub-gap energies. However, it should be noted that the optical transition rate of IB-CB is much lower than that of VB-IB. This can be understood in terms of optical transition theory in semiconductors. Based on the Fermi’s golden rule, the optical transition rate of VB-IB and IB-CB can be expressed as follows:

in which, $f_{C}\left(k\right)$, $f_{I}\left(k\right)$ and $f_{V}\left(k\right)$ represents the occupancy factors of electrons. From the Hall measurement, it is found that the Fermi level is still slightly below the energy level of IB states, which indicates that IB states are almost empty. It means that the occupancy factor of IB,$f_{I}\left(k\right)$, is very small. According to the above expression, the optical transition from VB to IB would be dominated while the probability of transitions from IB to CB is relatively small. It has been confirmed by the strong absorption at 1.8 eV and relatively week transition from IB to CB at 0.45 eV in Fig. S1.

**2. Demonstration of two-photon excitation processes in ZnTe:O with optically active IB states**

520

530

540

550

560

570

580

590

600

**PL Intensity (a.u.)**

**Wavelength (nm)**

ZnTe NBE

Excited by 633nm and 840nm lasers

(d)

(c)

(b)

(a)

Fig.S2. PL curves of ZnTe single crystal excited by 633 nm laser and 840 nm laser (a), sample S2 only excited by: 840 nm laser (b), 633 nm laser and 840 nm laser (c) and 633 nm laser with higher excitation power (×10) (d).

To investigate the nature of intermediate band states in ZnTe:O, it is essential to demonstrate the two photon absorption processes that correspond to the optical VB-IB and IB-CB transitions simultaneously. Fig. S2 illustrates the photoluminescence spectra of sample SC and S2 under excitation by lasers of 633nm or/and 844nm which correspond to the photon energies of 1.96 eV and 1.49 eV, respectively. For curve (a) in Fig. S2, the spectrum was recorded under excitation of both 633nm and 844nm lasers on the ZnTe single crystal. As discussed in the main manuscript, the ZnTe single crystal has a low density traps or defect states within the bandgap, and thus it is expected that no emission was observed in curve (a). For curve (b), the PLM processed sample S2 was excited only by 844nm laser. The corresponding photon energy is 1.49 eV, lower than the VB-IB transition of 1.8 eV. In this case, electrons in the VB cannot be excited into IB or CB, and thus no near band emission was observed as well. For curve (c), the PLM processed sample S2 was excited by two lasers simultaneously and a distinct near band emission was observed at 2.25 eV. It strongly suggested that two photon excitation takes place, in which, electrons were excited from VB to IB by 633nm laser (1.96eV) and the photo-excited electrons transit from IB to CB by absorbing photons from 633nm or 840nm. Thus, the photo-excited electrons in CB via two photon absorption give rise to the near-band emission of 2.25eV. Such up-frequency conversion process is a result of two photon absorption via intermediate band as a step-stone. As increasing the power density of 633nm, as shown in curve (d), the near band emission becomes stronger, indicating the enhanced two photon absorption within the ZnTe:O layer with optically active intermediate band states.

1. ^*^ Author to whom correspondence should be addressed. Electronic mails: [yejd@nju.edu.cn](mailto:yejd@nju.edu.cn) and [ffren@nju.edu.cn](mailto:ffren@nju.edu.cn) [↑](#footnote-ref-1)
